# A Treatise on the Diseases of the Eye

**Published:** 1842-12-24

**Authors:** 


### BIBLIOGRAPHICAL NOTICES.

*A Treatise on the Diseases of the Eye.* By W. Lawrence, F. R. S., etc., etc. From the last London Edition, with numerous additions, and fifty-seven illustrations. By Isaac Hays, M. D., etc. Philadelphia: 1842. 8vo. pp. 778.

Opthalmic medicine every where seems to have received a new impulse. For years Germany was preëminent in this important branch of pathology.

This was due to the establishment of a special chair of eye-medicine by the Empress Maria Louisa, in 1773. A complete revolution was thus effected; and England and France, whose surgeons had done so much in opthalmology, were completely eclipsed by the brilliant success of the eye clinique of Vienna. A long list of distinguished names attest the justness of the reputation of the German school—those of Barth, John Adam Schmidt, Beer, Rosas, Jaeger, Eble, Richter, Chelius, Ammon, Graefe, Juengken, and a host of others, are familiar to the surgical student. England and France have recently recovered from their apathy, and a number of excellent works have issued from the press of both countries. The treatises of Carron de Villars, Rognetta, Velpeau, Sichel, Vidal (de Cassis,) Furnari, &c., on opthalmic medicine, as well as the excellent contributions of the late Professor Sanson in the Dictionary, show the attention which the diseases of the eye receive at the hands of the French surgeons. Professor Velpeau gives annually a special course on this subject at La Charité, and Professor Sanson did the same at La Pitié. There are besides, the special private clinics of Sichel, Carron de Villars, Rognetta, and Zokalski. In Great Britain the standard works of Lawrence, Mackenzie, Middlemore, Guthrie, Travers, Tyrrell, &c., give substantial evidence of the interest there taken in the advancement of eye medicine. The ophthalmic infirmaries of London, Glasgow, and Birmingham, and the happy influence they exercise on the progress of science are well known.

The work before us is the fruits of the successful cultivation of eye-medicine in England. As the American editor justly remarks, its character "is too well established to require a word of commendation." The basis of this treatise was the Lectures which its distinguished author delivered in the London Eye Infirmary. It was first published in 1833, and the second edition, from which this is a reprint, in 1841. It shows a vast amount of erudition, and, at the same time, a practical familiarity with the subject, the result of extensive opportunities. The American editor has supplied some omissions in the Treatise, and he has added the results of his own experience in the treatment of most of the important diseases "derived from more that twenty years devotion to the subject, during all which period he has been attached to some public institution for the treatment of the Diseases of the Eye."

The work is cleverly printed, and handsomely illustrated.

